# Assessing the Credibility and Authenticity of Social Media Content for Applications in Health Communication: Scoping Review

**DOI:** 10.2196/17296

**Published:** 2020-07-23

**Authors:** Eva L Jenkins, Jasmina Ilicic, Amy M Barklamb, Tracy A McCaffrey

**Affiliations:** 1 Department of Nutrition, Dietetics and Food Monash University Notting Hill Australia; 2 Monash Business School Monash University Caulfield East Australia

**Keywords:** review, trust, social media, nutrition science, health, communication, health communication

## Abstract

**Background:**

Nutrition science is currently facing issues regarding the public’s perception of its credibility, with social media (SM) influencers increasingly becoming a key source for nutrition-related information with high engagement rates. Source credibility and, to an extent, authenticity have been widely studied in marketing and communications but have not yet been considered in the context of nutrition or health communication. Thus, an investigation into the factors that impact perceived source and message credibility and authenticity is of interest to inform health communication on SM.

**Objective:**

This study aims to explore the factors that impact message and source credibility (which includes trustworthiness and expertise) or authenticity judgments on SM platforms to better inform nutrition science SM communication best practices.

**Methods:**

A total of 6 databases across a variety of disciplines were searched in March 2019. The inclusion criteria were experimental studies, studies focusing on microblogs, studies focusing on healthy adult populations, and studies focusing on either source credibility or authenticity. Exclusion criteria were studies involving participants aged under 18 years and clinical populations, gray literature, blogs, WeChat conversations, web-based reviews, non-English papers, and studies not involving participants’ perceptions.

**Results:**

Overall, 22 eligible papers were included, giving a total of 25 research studies. Among these studies, Facebook and Twitter were the most common SM platforms investigated. The most effective communication style differed depending on the SM platform. Factors reported to impact credibility included language used online, expertise heuristics, and bandwagon heuristics. No papers were found that assessed authenticity.

**Conclusions:**

Credibility and authenticity are important concepts studied extensively in the marketing and communications disciplines; however, further research is required in a health context. Instagram is a less-researched platform in comparison with Facebook and Twitter.

## Introduction

### Background

Science, particularly the discipline of nutrition science, is currently facing credibility issues in the eyes of the general public [1,2]. Although nutrition science has contributed to countless discoveries and progressions in science, it is more complicated than other scientific disciplines in multiple ways. First, food is an essential part of every human’s life; thus, many people have a vested interest in nutrition and care greatly about their health [3]. Second, the significant research funding provided by the food industry creates conflicts of interest and often high levels of skepticism from the public [1,4,5]. Researcher Marion Nestle identified 76 industry-funded studies between March and October 2015, of which 70 reported results that were favorable to the sponsor’s interest, highlighting the potential bias in the industry [6]. Finally, there is an ongoing challenge to produce evidence-based science to facilitate recommendations that promote health on a population level, such as dietary guidelines [1].

The advent of the internet and, in particular, social media (SM; see Multimedia Appendix 1 for a glossary of terms) has permanently changed communication worldwide. Similar to traditional media (eg, newspapers, television, and radio), websites initially existed for one-way information dissemination through static web pages, known as *Web 1.0* [7]. However, progression to the second generation of the internet, *Web 2.0* (which includes SM), has facilitated a two-way interaction between users, creating a platform for collaboration, sharing, and socialization [7].

Currently, the use of SM in health interventions has had limited effectiveness, with participant engagement rates (Multimedia Appendix 1) being highly variable, ranging from 3% to 69% [8,9]. Health promotion organizations have maintained a one-sided communication approach, sharing serious and factual messages across their platforms, often reaching only a limited audience [10,11]. In contrast, product and corporate brands (Multimedia Appendix 1) have effectively adapted their marketing strategies to utilize the features of SM by being more likely to use hashtags to increase the reach of their posts, interact with their followers to create a two-way communication channel, and run promotions such as competitions to encourage user engagement [10]. Many large and well-known corporations such as soft drink companies are faceless and do not have a permanent ambassador and instead utilize celebrity endorsers (Multimedia Appendix 1) periodically to promote their products and improve their image and reputation [12].

Similar to celebrity endorsers, SM influencers (SMIs) or “individuals or groups of individuals who can shape attitudes and behaviours through online channels,” are arguably human brands (Multimedia Appendix 1), which often enable consumers to see them as regular individuals with whom they share common values [11,13]. SMIs are often referred to as microcelebrities (Multimedia Appendix 1) as they provide insight into their private lives and create the perception that they are constantly accessible and intimately invested in their audience [14]. The number of people that connect and engage with posts by SMIs is high, as their audience consists of like-minded people, allowing brands that partner with them to target specific demographic and lifestyle groups [15]. In contrast, nutrition professionals (Multimedia Appendix 1), who typically promote evidence-based science on SM, must maintain a sense of professionalism online and, therefore, cannot create the same type of content without risking their career prospects [16].

In the existing posttruth era (Multimedia Appendix 1), science experts (including nutrition professionals) are often less highly regarded, and emotional appeals are often the most effective methods of communication [2,17]. Consumers are frequently turning to celebrities and SMIs (who hold no formal qualification) for health and lifestyle advice, creating many implications for their health and well-being [18,19]. In particular, health and wellness advice spread on online platforms tends to be misinformation rather than evidence-based information [20]. For example, the *A-list* celebrity Gwyneth Paltrow has consistently been in the public eye after controversial and dangerous health claims were made by her health and wellness brand, *Goop* [21,22]. Numerous articles on *Goop’s* website and Instagram promote detoxifying the body (a process that is naturally performed by the liver) and cutting out essential food groups from the diet (eg, carbohydrates) [23]. These recommendations are based on anecdotal evidence and pseudoscience, perpetuating disordered eating habits and nutritional imbalances [24].

In the marketing literature, underlying factors such as the endorser’s expertise, trustworthiness, attractiveness, and authenticity have been shown to influence people’s behavior in traditional forms of advertising (eg, television commercials, celebrity partnerships) [25,26]. This review was focused on various theories and models from psychology literature that draw on the concepts of expertise, trustworthiness, and authenticity: self-determination theory (SDT; Multimedia Appendix 1), source credibility model (Multimedia Appendix 1), and the elaboration likelihood model (ELM; Multimedia Appendix 1). The following section (*Theoretical Framework*) summarizes these concepts and their nexus.

### Theoretical Framework

Authenticity is the concept of “being true to the self in terms of an individual’s thoughts, feelings, and behaviours reflecting their true identity” [27]. SDT encompasses the concept of authenticity with 3 primary components: autonomy, competence, and relatedness [28]. SDT posits that authenticity involves an individual’s engagement in intrinsically motivated behaviors, *behaviors that come from a person’s innate desires and passions* [28]. In marketing literature, individuals tend to perceive another person (eg, a celebrity) as authentic when the other person’s actions reflect his or her autonomous, self-determining, true self [26]. Celebrities who are perceived as authentic have a higher level of influence over others, both online and offline [29]. Many young people do not verify information found online, leaving them particularly susceptible to celebrity influence [30]. Therefore, exploring the factors that impact authenticity on SM could be useful to inform health communication and behavior change campaigns.

The source credibility model suggests that a credibility judgment is determined based on the source’s (eg, a celebrity’s) expertise, trustworthiness, and attractiveness [31]. The 3 dimensions of source credibility differ: (1) expertise refers to the perceived knowledge and education level of the source, (2) trust refers to the listener’s confidence in and level of acceptance of the speaker or message, and (3) attractiveness refers to the perceived physical attractiveness of the source. As a credible message is influential, many individuals and brands place a high level of importance on creating and maintaining credibility [32]. Typically, SMIs are perceived as credible as they are physically attractive and share aesthetically pleasing photos relevant to their field of perceived expertise (eg, health) to showcase their desirable lifestyle [33]. Similarly, large corporations utilize attractive and trustworthy celebrity endorsers to be the face of their marketing campaigns to increase the brand’s credibility [25]. More recently, the use of SM platforms for health advice has made it difficult for laypeople to differentiate a credible, evidence-based message from a noncredible message in an environment where everyone appears to have expertise [34].

Message content is assessed through cognitive processing (Multimedia Appendix 1), explained by the ELM (Multimedia Appendix 1). The ELM describes how people manage the information they encounter and the way in which it influences attitude change [35,36]. There are 2 processing routes: central and peripheral [35,36]. This review focuses on the peripheral route of processing, where messages are evaluated using heuristics as there is low motivation to critically evaluate the content of a message [37]. On SM, heuristics are commonly used to assess the credibility of a message, for example, using celebrity endorsers to promote a product triggers familiarity and infers the product’s credibility by association. Before Facebook (Australia only) and Instagram trialed the removal of the number of publicly visible likes on a post, consumers assessed credibility through bandwagon heuristics (Multimedia Appendix 1), triggered by a mass of user opinion (eg, seeing a high number of comments on a SM post) [38]. The herd mentality (Multimedia Appendix 1) of liking what others like arises through the process of status-seeking and the need to be associated with others [39]. Another common way for consumers to make prompt judgments is via the expertise heuristic, cued when a consumer sees an official authority (such as an organization) as the source of information, whether it is an SM post, a news article, or a website [40]. By quickly associating an organization or individual as an expert source, less motivation is required to assess source credibility.

### Purpose and Objective

Previous reviews have assessed the credibility of health information online, finding that factors such as clear website layout and professional design increased credibility [41-43]. However, much of this research is not applicable to SM platforms, which are often less curated and focus on fast-paced status updates.

As SM is a relatively new area of research, a scoping review was considered the most appropriate method to explore the topic area. Our research in applying social marketing techniques to the field of nutrition has led us to recognize the importance of using marketing and communication techniques, particularly on SM [9,11,44]. The aim of this scoping review was to understand and explore the factors that affect consumers’ perceptions of message and source credibility (ie, expertise and trustworthiness) and authenticity on SM platforms to better inform nutrition science SM communication best practices. A secondary objective was to examine the fields that are currently undertaking this type of research and the theories used to inform the research.

## Methods

### Search Strategy and Databases

The Preferred Reporting Items for Systematic Reviews and Meta-Analyses Scoping Review Checklist and the Joanna Briggs Institute Reviewer’s Manual were used throughout the review process [45,46].

Key databases from health, psychology, and business disciplines were used to conduct the final search in conjunction with a university librarian on March 27, 2019. Cumulative Index of Nursing and Allied Health Literature (CINAHL) Plus (422 results), Scopus (2199 results), Excerpta Medica dataBASE (EMBASE; 697 results), Ovid Medical Literature Analysis and Retrieval System Online (MEDLINE; 326 results), PsycINFO (1223 results), and Business Source Complete (1375 results) were searched for title, abstract, and keywords to identify the initial 6242 articles (example search strategy provided in Multimedia Appendix 2). All articles were imported into Covidence online software (Veritas Health Innovation) to manage the reviewing process. Manual searches were conducted by checking the reference lists of included studies to identify additional papers that the search may have missed; however, no papers were found.

### Inclusion and Exclusion Criteria

**Figure 1:**
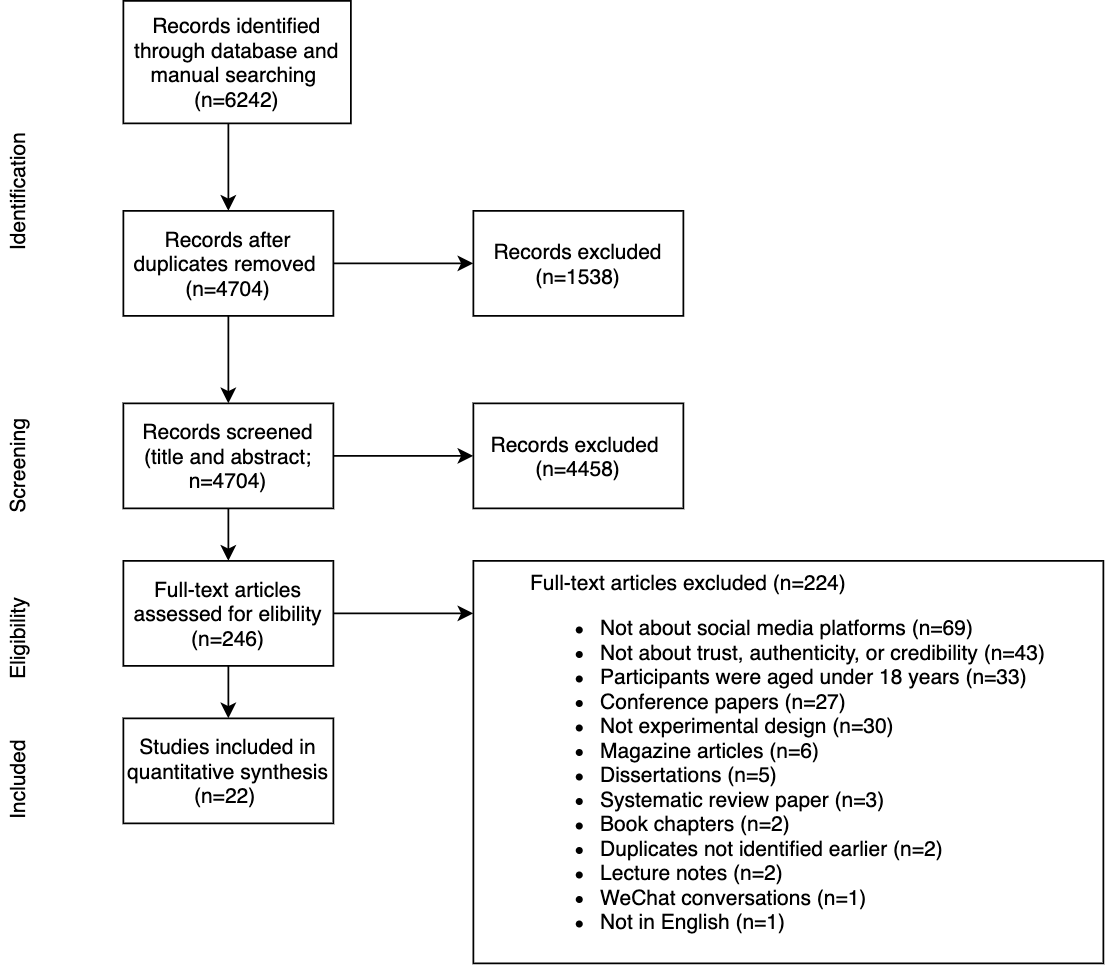
Preferred Reporting Items for Systematic Reviews and Meta-Analyses for Scoping Reviews flow diagram of the search and study selection process.

The inclusion criteria were experimental studies (ie, stimuli provided), studies focusing on microblogs (eg, Facebook, Twitter; Multimedia Appendix 1), studies focusing on healthy adult populations (as clinical populations often use SM to search for very specific health information), and studies focusing on source credibility or authenticity. No date restrictions were used. Exclusion criteria were studies involving participants aged under 18 years and clinical populations, gray literature, blogs, WeChat conversations, web-based reviews, studies that did not involve participants’ perceptions, and non-English papers (Figure 1).

### Screening

Overall, 2 investigators (ELJ and AMB) independently screened the title and abstract of the included papers against the inclusion and exclusion criteria. This process was repeated for full-text screening, with any conflicts being discussed until a joint consensus was reached. There were 22 final papers (Figure 1).

### Data Extraction

Data extraction was conducted independently by a researcher (ELJ) using Microsoft Excel 2019 (Microsoft Corporation) and was then cross-checked by a fellow researcher (AMB). Following data extraction, the results were collated based on various parameters such as SM platform focused on manipulation stimuli, outcome, and scales used for data collection. All papers were given a category to summarize their topic area, such as *celebrity*, *political*, *marketing*, *health*, or *general* (for studies that assessed source credibility without an overarching theme).

## Results

### Key Characteristics

Of the 22 papers that met the inclusion criteria, 3 papers reported on 2 separate studies, giving a total of 25 research studies [47-49]. The number of participants in the included studies ranged from 85 to 3476 [49,50]. Most studies were conducted in the United States (n=16) and had between-subject experimental designs (n=19). All studies used convenience sampling, excluding one that used random sampling [51]. Many studies (n=19) had no inclusion or exclusion criteria for recruitment, whereas only 2 studies specified exclusion criteria that required the participants to have an active SM account [52,53]. Student populations were predominant (n=17), where college students participated in exchange for course credit. Paid online participants were recruited in 5 of the studies via either Mechanical Turk (an Amazon platform) or professional market research companies [50-52,54,55]. Generally, the student population had a lower average age than the paid participants. Demographic data were not reported in 3 studies [56-58].

A total of 4 microblogging platforms were used: Twitter (n=10), Facebook (n=8), Instagram (n=3), and YouTube (n=3; Multimedia Appendix 1). Furthermore, 2 papers focused on both Facebook and Twitter [50,59]. Credibility was the most reported outcome (n=18), followed by trust (n=4); authenticity was not reported in the included studies. The predominant fields of research included communications (n=11), psychology (n=4), and marketing (n=2). The source credibility model (n=13) and the modality, agency, interactivity, and navigability (MAIN) model (n=7) were frequently used to inform research across disciplines. Other theories included social capital theory (n=2) and self-disclosure theory (n=2). The most commonly manipulated variables were the number of likes (n=4), source of the post (n=4), number of followers (n=3), and number of retweets (n=3).

### Scales

All papers used a scale to assess credibility, with the most common being McCroskey and Teven’s Source Credibility Scale (n=6; α=.94) and Ohanian’s Source Credibility Scale (n=4; α>.8) [31,60]. McCroskey and Teven’s validated scale, used to assess the credibility of political and public figures, includes 3 constructs: goodwill, trustworthiness, and competence [60]. Ohanian’s validated scale, created to assess the credibility of celebrity endorsers, also includes 3 constructs: attractiveness, trustworthiness, and expertise [31]. Other studies created their own scales for data collection, sourcing items from the literature, and were not validated before use [47,51,61,62].

### Main Observations

Most papers (n=15) involved the manipulation of text (eg, in a SM post) via Facebook or Twitter feeds to assess credibility (n=11), trust (n=4), or both (n=2). Studies primarily explored the tone of voice, bandwagon cues, and expertise [47-49,51,56,58,59,63-66]. Full details of the studies are reported in Multimedia Appendices 3-7.

#### Language Use

Overall, 3 papers included in the review assessed message credibility: how message characteristics impact credibility perceptions (Multimedia Appendix 1). In a study conducted on Facebook with a student sample (mean age 19 years), language usage was found to impact credibility judgments, with a gain-framed post (focusing on the benefits of exercise) eliciting positive emotions and increasing credibility when compared with a loss-framed post (focusing on the risks of not exercising; Tables 1 and 2; Multimedia Appendix 3) [47].

On Twitter, Houston et al [51] found that the tone of voice impacted credibility; nonopinionated tweets (written as a headline), which conveyed no personal opinion, were more credible than opinionated tweets that used humor or sarcasm and conveyed strong personal opinion among consumers aged over 18 years, participating for monetary rewards (Tables 1 and 2; Multimedia Appendix 4).

Similarly, Yilmaz and Johnston [59] compared language framed with personal experiences to depersonalized language (ie, factual and data-based) on Facebook and Twitter in a sample of 257 students (Tables 1 and 2; Multimedia Appendix 5). Differing results were found among the SM platforms; personalized Facebook posts were more competent and trustworthy than personalized tweets [59]. However, depersonalized tweets were more competent and trustworthy than depersonalized Facebook posts [59]. Thus, personalized language was an effective way to increase credibility on Facebook but not on Twitter [59].

**Table 1:** Main findings of included papers and their effect on credibility or trust, separated by manipulated variable: number of likes, number of followers, number of retweets, source, and language.

Outcomes/author (year) [citation]		Population group	Key significant results
**Number of likes**			
	Borah and Xiao (2018) [47]	Students	In the 2 studies conducted, the number of likes did not affect source credibility overall when looking at Facebook posts (study 1: *P*=.93; study 2: *P*=.09)
	Phua and Ahn (2016) [66]	Students	Brand trust was higher when likes were high on Facebook post (*P*<.005) or when friends’ likes were high (*P*<.001). Friends’ likes were more important in trust than overall total likes (*P*<.005). The number of likes had no direct effect on brand trust when the intensity of Facebook use was controlled for (*P*=.89)
	Shen et al (2019) [50]	Paid online workers	Bandwagon cues did not impact credibility when looking at images on Twitter and Facebook (*P*=.85)
**Number of followers**			
	Jin and Phua (2014) [48]	Students	A higher number of Twitter followers on the celebrity’s account increased source credibility and intention to build an online friendship with the celebrity endorser for all dimensions of source credibility: physical attraction (*P*<.05), trustworthiness (*P*<.05), and competence (*P*<.01)
	Lee (2018) [49]	Students	The number of followers on Facebook made a statistically significant difference on the believability of the answer (*P*<.05), with a high number of followers increasing believability. There were no significant results for trustworthiness or accuracy
	Westerman et al (2011) [57]	Students	Trustworthiness indicated an inverted U-shaped relationship with the number of followers on Twitter (*P*=.02)
**Number of retweets**			
	Lin and Spence (2018) [63]	Students	The highest level of trust (on Twitter) was when participants viewed the post with 400 retweets, followed by 40 retweets, whereas 4000 retweets had the lowest level of trust (*P*=.01). Participants perceived the highest levels of source competence when viewing the post with 40 retweets, followed by 400 retweets. The post with 4000 retweets had the lowest perceived competence (*P*=.01)
	Lin and Spence (2019) [64]	Students	There were significant differences in trust perceptions across varying retweet conditions (*P*=.046). People who viewed the FDA’s^a^ Twitter page containing 4000 retweets were more likely to perceive lower organizational trust than the condition of 40 retweets (*P*<.05)
	Lin and Spence (2016) [65]	Students	Participants perceived lowest competence when viewing a peer’s Twitter page with no retweets (*P*<.001). The highest level of perceived source goodwill, trustworthiness, and competence was when participants viewed the CDC^b^ page with no retweets (*P*<.001)
**Source (expert, peer, or stranger)**			
	Borah and Xiao (2018) [47]	Students	In the 2 studies conducted on Facebook, the CDC and WebMD authors were seen as more credible than unknown authors (study 1: *P*<.01; study 2: *P*<.01)
	Lin and Spence (2018) [63]	Students	Participants viewing an FDA expert’s Twitter account were more likely to perceive higher trust (*P*=.01), competence (*P*<.001), and goodwill (*P*<.001) than those viewing a peer or stranger’s account
	Lin and Spence (2016) [65]	Students	Higher credibility was assigned to risk information from an expert compared with a peer and a stranger on Twitter (*P*<.001)
**Language (message credibility)**			
	Borah and Xiao (2018) [47]	Students	In the 2 studies conducted, a gain-framed message was more credible than a loss-framed message on Facebook (study 1: *P*<.001; study 2: *P*<.001)
	Houston et al (2018) [51]	Paid workers	Nonopinionated tweets were perceived as more credible than opinionated tweets (*P*<.001)
	Yilmaz and Johnson (2016) [59]	Students	Personalized status updates on Facebook were seen as more competent and trustworthy than personalized tweets (*P*=.007, *P*=.001 respectively). Depersonalized tweets were more trustworthy than the source of depersonalized status updates on Facebook

^a^FDA: Food and Drug Administration.

^b^CDC: Centers for Disease Control and Prevention.

**Table 2:** Included papers, main outcomes, and the effect on either brand trust, message credibility, or source credibility (including trustworthiness, believability, and competence) as specified in their results.

Factors		Platform	Population	Outcome	Result^a^	Relevant papers
**Language use^b^**						
	**Gain-framed language**					
		Facebook	Student	Message credibility^b,c^	Increase	Borah and Xiao [47]
	**Personalized language**					
		Twitter	Student	Competence and trustworthiness	Decrease	Yilmaz and Johnson [59]
		Facebook	Student	Competence and trustworthiness	Increase	Yilmaz and Johnson [59]
	**Exposure to civil discussion**					
		Facebook	Student	Trustworthiness	Increase	Antoci et al [67]
	**Nonopinionated language**					
		Twitter	Paid worker	Message credibility	Increase	Houston et al [51]
		YouTube	Student	Message credibility	No effect	Zimmermann and Jucks [68]
**Bandwagon heuristics^b^**						
	**High number of likes**					
		Facebook	Student	Source credibility and trustworthiness	No effect	Borah and Xiao [47]
			Student	Brand trust	Increase	Phua and Ahn [66]
	**High number of followers**					
		Facebook	Student	Believability	Increase	Lee [49]
		Twitter	Student	Source credibility	Westerman and Spence: unclear; Phua and Ahn: increase	Westerman and Spence [57]; Phua and Ahn [66]
	**Narrow ratio of the number of followers to the number of follows**					
		Twitter	Student	Competency	Increase	Westerman and Spence [57]
	**High number of retweets**					
		Twitter	Student	Trustworthiness	Decrease	Lin and Spence [63-65]
	**High number of friends**					
		Facebook	Student	Believability and trustworthiness	Increase	Lee [49]
**Expertise heuristic^b^**						
	Post from expert source	Facebook and Twitter	Student	Source credibility	Increase	Borah and Xiao [47]; Lin and Spence [63,65]
**Other^b^**						
	Interaction with followers	Twitter	Student	Source credibility	Increase	Jahng and Littau [62]
	High perceived privacy control	Facebook	Student	Trust	Increase	Antoci et al [67]
	Positive brand attitude	Instagram	Paid worker	Brand credibility	Increase	De Veirman and Hudders [52]; Jin and Muqaddam [55]
	Prosocial attitude online	Twitter	Student	Source credibility	Increase	Jin and Phua [48]
	Recency of updates (frequent)	Twitter	Student	Source credibility	Increase	Westerman and Spence [56]
	Snapshot aesthetic (vs studio aesthetic)	Instagram	Paid worker	Brand credibility	Increase	Colliander and Marder [54]
	Preexisting photoshop/internet skills (when looking at photoshopped images)	Twitter and Facebook	Paid worker	Source credibility	Decrease	Shen et al [50]
	Ethos message appeal (compared with logos and pathos)	YouTube	Student	Source credibility	Increase	English et al [61]
	Consumer-generated advertising (compared with firm-generated advertising)	YouTube	Student	Source credibility	Increase	Lee et al [69]
	Caucasian ethnicity (compared with African American)	Facebook	Student	Source credibility	Increase	Spence et al [58]

^a^On the basis of reported results from studies summarized in Multimedia Appendices 3-7.

^b^For further context, explanation, and examples of these factors, refer to Multimedia Appendices 3-7.

^c^Credibility comprises trustworthiness, expertise, and sometimes attractiveness, depending on the individual paper.

#### Bandwagon Heuristics

Bandwagon cues such as the number of followers, number of retweets, and number of likes were a way in which student participants (mean age range 19-22.9 years) assessed source credibility across 30% (8/22) of the included papers; however, the findings were inconsistent among studies [47-49,56,63-66].

Borah and Xiao [47] assessed the number of likes on a Facebook post (150 likes or 2 likes) and found that the manipulation had no significant effect on credibility or trust levels (Tables 1 and 2; Multimedia Appendix 3). This differed from the study by Phua and Ahn [66], which found that brand trust increased when overall likes or friends’ likes on the post were higher (Tables 1 and 2; Multimedia Appendix 3). Similarly, Lee’s experiment [49], which involved a question-and-answer format on a Facebook post, found that when the source had a higher number of followers, the answer posted was rated as more believable than an answer posted by a source with a lower number of followers (Multimedia Appendix 3). However, this relationship was not observed for the accuracy or trustworthiness dimensions of source credibility. Lee’s second experiment assessed the credibility of a source with a high number of friends compared with a low number, finding that the source with more friends was seen as more believable and trustworthy (Multimedia Appendix 3) [49].

On Twitter, Westerman et al [57] found no linear relationship between the number of followers and credibility. In fact, too many followers (n=70,000) or too few followers (n=70) reduced the level of trust compared to those with 7000 followers, who had the greatest level of trust (Multimedia Appendix 4) [57]. However, Jin and Phua [48] reported that a higher number of followers (n=14,677,050) increased source credibility (Tables 1 and 2; Multimedia Appendix 4). The ratio of the number of followers to the number of follows on Twitter provided a cue for participants to assess credibility, with a narrow gap (ie, similar ratio of Twitter followers to follows) being perceived as more competent than a wide gap (ie, more Twitter followers compared with follows; Multimedia Appendix 4) [57]. High numbers of retweets (from other Twitter profiles) reduced credibility in 3 studies [63-65]. When using an organization’s Twitter page (*Centers for Disease Control and Prevention* [CDC] or *Food and Drug Administration* [FDA]), a post with 4000 retweets was found to be less trustworthy and competent than posts with 40 or 400 retweets (Table 1; Multimedia Appendix 4) [63-65].

#### Expertise Heuristics

Manipulating the source of the Facebook or Twitter posts was found to impact source credibility in 3 studies with student participants [47,63,65]. Expert sources, such as the CDC*,* were perceived as more credible than strangers when disseminating health information in Facebook status updates (Multimedia Appendix 3) [47].

Similarly, expert sources (eg, FDA) were considered more trustworthy and competent and, thus, overall more credible than strangers or peers on Twitter (Tables 1 and 2; Multimedia Appendix 4) [63,65]. Jin and Phua [48] assessed celebrity source credibility by manipulating a news story of the celebrity (shared on Twitter) to be either prosocial (donating to a charity) or antisocial (drug abuse; Multimedia Appendix 4). Consumers were more likely to identify with prosocial celebrity and perceive them as more credible, suggesting that reputation can affect credibility perceptions [48].

## Discussion

### Principal Findings

Surprisingly, there were no studies in the field of health and nutrition research included in this scoping review; however, there are some important learnings that could be utilized for nutrition and health communication. There were many different factors that affected the perceived credibility of a message and source on SM, such as language usage, expertise heuristics, and bandwagon heuristics. However, no information was found on the factors affecting perceived authenticity in this context. The scales used as well as the different models and theories to inform various fields of research are reported.

### Language Usage

The results of the included studies indicated that the language used in tweets affected message credibility. Personalization was more effective on Facebook, whereas depersonalization was more effective on Twitter [59]. This can be explained by the differing functions of these platforms. The audience of a tweet is largely unknown as the public nature of Twitter allows anyone with or without an account to view a profile (provided the profile is not locked) [70]. Having a large and primarily unknown audience creates a challenge to balance self-expression and impression management; users want to share information but do not want to be negatively judged [70]. The fear of other people’s negative opinions and judgment makes it easier to post factual, depersonalized tweets with no personal opinion involved [70]. Furthermore, the linguistic style of Twitter differs from other SM platforms because of the 280-character (previously 140) limit for tweets [59]. When users share long personal stories, it differs from the concise updates that are typically shared on Twitter, reducing the contextual appropriateness of the information [59]. This differs from Facebook, which has an extremely high character limit for status updates (63,206 characters) and requires both parties to accept a friend request before viewing each other’s content (provided the privacy of the page is not set to public) [59]. Thus, when a personal status update is made on Facebook, the recipients are generally people who have an existing relationship with the user and an established level of trust, instantly increasing the credibility of the post [59]. Therefore, message characteristics such as language style and the platform being used should be considered by health professionals when creating SM posts.

### Expertise Heuristics

Each study in the review that compared an expert to a stranger or peer found that the expert was more credible [47,63,65]. This is explained by the expertise heuristic, which is triggered when a consumer sees an official authority as a source [71]. For example, when tweets were posted from the CDC’s Twitter account, they were seen as more credible than when an unknown person tweeted [65]. These findings are consistent with the existing literature on website credibility, whereby listing an author’s affiliations, credentials, or qualifications triggers the expertise heuristic and increases the credibility of the information or message presented [72,73]. In addition, a recent review found that websites run by health institutions (such as the CDC) were considered more trustworthy than private websites [42]. Health practitioners should include their credentials (relevant to their field of research) on their SM page, so that their expertise is clear to the audience and credibility can be established.

### Bandwagon Heuristics

A key focus of the included papers was bandwagon heuristics, which relate to the number of likes, followers, or retweets assigned to information on SM [71]. It is known that people base their own decisions on other people’s endorsements and opinions, particularly when purchasing products [74]. Typically, a bigger bandwagon (ie, a high number of likes, comments, or shares) will result in a greater perception of source credibility [40,75]. However, the reviewed papers that manipulated bandwagon cues provided differing results than expected; a high number of retweets (n=4000) had the lowest perceived trustworthiness of the conditions, and two papers found no difference in credibility between the low and high like manipulations (Multimedia Appendices 3 and 4) [47,50,63,64]. Some consumers perceived popular content as having lower credibility, described as a *reverse bandwagon heuristic* (Multimedia Appendix 1) or the *snob effect* (Multimedia Appendix 1) [39,76]. This arises from the consumer’s need to identify as an individual in society, without conforming to social norms [39,76]. Some people do not follow trends and are not willing to follow others without independently thinking and making their own judgments. The *snob effect* causes a deviation from the norm (ie, liking the post) and, thus, when a consumer sees a high amount of engagement on SM, it can result in negative opinions of the source and information presented, reducing the perceived credibility [63]. However, in 2019, attempting to reduce the pressure people feel when they post online, Instagram (globally) and Facebook (in Australia) trialed removing the number of likes on posts so that likes are no longer publicly visible to others [77]. Rather than displaying the number of people who have liked the post, the display now shows one user who has liked the post followed by the phrase “and others” [77,78]. Therefore, in the future, the use of bandwagon cues to judge credibility may be less prevalent as the engagement of a post can no longer be viewed publicly in a numerical format [77].

### Scales

The most commonly used scale in the research papers was McCroskey and Teven’s Source Credibility Scale (n=6), used to assess the credibility of a source of information (ie, a person). In contrast, message credibility was assessed by examining the characteristics of a message such as the structure, perceived accuracy, and language used [79]. Flanagin and Metzger’s Scale of Message Credibility Online (2013) assesses 5 dimensions of credibility—believability, accuracy, trustworthiness, bias, and completeness—and was utilized in 2 papers [49,50]. Other papers that assessed message credibility adapted their own scale from multiple sources in the literature to evaluate the language used in Facebook posts, tweets, or message appeals in YouTube videos [47,51,61]. Internal reliability was assessed with Cronbach alpha values at an acceptable level (α>.7) in all three papers that adapted their own scale. However, there was no exploratory or confirmatory factor analysis conducted, creating implications for the validity and generalizability of the research.

### Fields of Research

Communications and psychology were the predominant fields of research within the included papers. The theories and models used to underpin research overlapped among disciplines, with the source credibility model being the most common within communications, information research, psychology, and business. The MAIN model (a digital extension of source credibility model) was also used frequently in communications, information research, computer science, and psychology. Previously, source credibility and its impact have been investigated more broadly in the health discipline to assess consumers’ perceptions of health information online, but this was limited to websites and did not include SM [80]. To our knowledge, health communication research has not utilized the MAIN model or source credibility model. Using these models to inform research in different fields resulted in the perception of credible spokespeople on SM. Thus, these models should be utilized to increase the effectiveness of health communication within nutrition research.

### Gaps in Knowledge

On the basis of the search strategy and the inclusion and exclusion criteria, the concept of authenticity has not been explored in this specific context. In addition, there was a lack of research regarding SMIs or nutrition professionals as the source of SM posts. Most studies focused on organizations or an unknown fictional author as the source. As SMIs are key to digital marketing and health communication, research into consumer perceptions of the source credibility and authenticity of SMIs would be beneficial to further understand how they communicate effectively with their followers. Results from these future studies would be beneficial for informing the delivery of health communication in various digital formats.

### Strengths

The strength of the papers included in the scoping review was the large sample size that was achieved (range of the number of participants from included studies was 85-3476). As most papers had a student cohort participating for university credit, large numbers of participants were recruited. In addition, many of the papers completed a manipulation check during the pilot of the survey to ensure that the mock scenario they were creating was robust, for example, testing if the *high retweet* condition of 4000 retweets was actually considered *high* by the participants, limiting the confounding factors that could arise if the manipulations were perceived differently than intended [63].

### Limitations

As most papers (n=21) used convenience sampling, selection biases were inherent. Furthermore, generalizability was limited to the geographical area in which the research was conducted, making it difficult to draw conclusions without conducting further research. Student samples were predominant (n=17), further limiting the variability and generalizability of results as student samples (referred to as western, educated, industrialized, rich, and democratic [WEIRD]) [81] are seen as more homogenous in terms of education level and socioeconomic status than the general public [81,82]. Furthermore, cross-sectional questionnaires were used as the method of data collection, which is self-reported, adding a level of bias to the results as participants can be deceiving intentionally or unintentionally [83]. A methodological limitation of undertaking a scoping review is the omission of quality appraisal of studies, usually conducted during data extraction in a systematic literature review.

### Conclusions and Recommendations

Fostering credibility online should be considered by corporate and human health brands to create a stronger relationship with their audience. This scoping review highlighted that message and source credibility can be affected by language usage, expertise heuristics, and bandwagon cues. Gaps in the literature were identified, highlighting the need for further research on SM platforms, as Instagram and YouTube were studied less than Facebook and Twitter. The main field of research identified from the included papers was communications, with no papers from health or nutrition science. Currently, there is a limited understanding of the use of SMIs and science experts to relay health messages. Further research needs to be undertaken to apply information from communications (on source and message credibility and authenticity) in a health context and in populations other than students.

## Appendix

Multimedia Appendix 1 Glossary of terms.

Multimedia Appendix 2 Search strategy (Scopus database).

Multimedia Appendix 3 Research studies assessing trust and credibility on Facebook.

Multimedia Appendix 4 Research studies assessing trust and credibility on Twitter.

Multimedia Appendix 5 Research studies assessing credibility on both Facebook and Twitter.

Multimedia Appendix 6 Research studies assessing credibility on Instagram.

Multimedia Appendix 7 Research studies assessing credibility on YouTube.

## Abbreviations

CDC: Centers for Disease Control and Prevention
ELM: elaboration likelihood model
FDA: Food and Drug Administration
MAIN: modality, agency, interactivity, and navigability
SDT: self-determination theory
SM: social media
SMI: social media influencer
