# Supplementary material for: Assessing the Credibility and Authenticity of Social Media Content for Applications in Health Communication: Scoping Review
Source: J Med Internet Res. 2020 Jul 23;22(7):e17296. doi: 10.2196/17296 (PMC7413282; doi:10.2196/17296)
Supplement: Multimedia Appendix 1 [file jmir_v22i7e17296_app1.docx]

# Multimedia appendix 1: Glossary of terms

A glossary of terms is provided to explain terms used in health and marketing that may be unfamiliar when used outside of either discipline.

| ***Term*[reference]** | ***Definition*** |
| --- | --- |
| Ad scepticism [1] | The tendency to disbelieve the informational claims of advertising. |
| Bandwagon heuristic/effect [2] | Implies collective endorsement and popularity of the underlying content. The idea that ‘if others think that something is good, then I should too’. |
| Brand attitude [3] | The positive or negative feelings and opinions of consumers toward a brand. |
| Celebrity [4] | A famous person, especially in entertainment or sport. |
| Cognitive processing/elaboration [5] | The use of pre-existing knowledge in support of new learning i.e. relating new information to something you already know. |
| Consumer-generated advertising [6] | Brand content created by consumers, such as a picture, video, or blog post about a product, often shared over social media. |
| Corporate brand [7] | Has a broader scope than promoting products and services. The brand name is used in advertising to stakeholders. Importance is placed on the attitude and spirit behind the company e.g. Apple, Nike. |
| Elaboration Likelihood Model [8]   - Central processing - Peripheral processing | A model that proposes every message is undergoing the process of persuasion, affecting the attitude and views of the reader. There are two routes to persuasion, equally effective but used at different times:   - Central route: consists of thoughtful consideration of the arguments (ideas, content) of the message. The consumer is being an active participant in the process of persuasion. Requires motivation and ability. - Peripheral route: When the consumer decides whether to agree with the message based on other cues besides the strength of the arguments or ideas in the message. When there is little motivation to think critically. |
| Engagement [9] | Refers to social media activities that include browsing, liking, commenting on, and sharing content. |
| Facebook [10] | A social networking website where users can post comments, share photographs, and post links to news or other interesting content on the web, chat live, and watch short-form video. |
| Herd behaviour [11] | The phenomenon of individuals deciding to follow others and imitating group behaviours rather than deciding independently on the basis of their own, private information. |
| Human brand [12] | Any well-known persona who is the subject of marketing communication efforts. Refers to both SMI and celebrities. |
| Instagram [13] | A social networking site that involves posting photos with the option of using enhancement filters, and nonreciprocal following of other users. Accounts can be private (request to follow others) or public (anyone can follow). |
| Message credibility [14] | How message characteristics (e.g. language intensity, message content, message delivery) impact perceptions of believability, either of the source or the source’s message. |
| Microblogging platforms [9] | A type of blog in which users can post small pieces of digital content like pictures, video, or audio on the Internet. |
| Micro-celebrity [15] | People “amping up” their popularity over the Web using techniques like video, blogs, and social networking sites (e.g. Instagram, Facebook). |
| Nutrition Professional [16] | An individual who holds a degree in Nutrition, with 3+ years’ experience working within the field. |
| Post-truth era [17] | Relating to a situation in which people are more likely to accept an argument based on their emotions and beliefs, rather than one based on facts. |
| Reverse bandwagon heuristic/effect [18] | Consumer perceives the lowest credibility (or other outcome) when the bandwagon is highest (e.g. number of followers). |
| Self-Determination Theory [19]   - Autonomy - Competence - Relatedness | A theory of human motivation concerned with people’s innate psychological needs. Concerned with the motivation behind people’s choices that occur without external influence.   - Autonomy: person’s need to feel that his or her activities are self-chosen, self-governed, and self-endorsed. - Competence: A person’s innate, life-span tendency to seek feelings of effectiveness, achievement, and challenge in his or her activities. - Relatedness: A person’s need to feel a sense of closeness with others. |
| Snob effect [20] | In economics: Snob consumers see the price as an indicator of privilege and avoid using popular brands in order to have an inner-directed consumption experience, i.e. the need to be the only consumer of a product.  On social media: When consumers see others have liked/engaged with a post and, therefore, do not want to engage as they do not want to be associated with others. |
| Social media [21] | Any web-based communication channel, dedicated to community-based input, interaction, content-sharing, or collaboration. |
| Social media influencer [21] | Individuals, or groups of individuals, who can shape attitudes and behaviours through online channels. Can be a celebrity or someone that is unknown outside of social media. |
| Source credibility [22] | A communicator’s positive characteristics that affect the receiver’s acceptance of a message. Includes the speaker’s attractiveness, trustworthiness, and expertise. |
| Twitter [23] | An online news and social networking site where people communicate in short messages called Tweets. There is a 280-character limit. |
| YouTube [24] | A public video-sharing website where people can experience varying degrees of engagement with videos, ranging from casual viewing to sharing videos in order to maintain social relationships. |

## Reference List

1. Obermiller C, Spangenberg ER. Development of a scale to measure consumer skepticism toward advertising. J Consum Psychol. 1998;7(2):159-86.[doi: 10.1207/s15327663jcp070203].

2. Shyam Sundar S. The MAIN Model: A heuristic approach to understanding technology effects on credibility. 2008 [cited 2019 April 4]. Available from: <https://pdfs.semanticscholar.org/de80/aa094f380342a632eadb0ee8d4221e8920ba.pdf>

3. Çadırcı TO, Gungor AS. Gap between mobile and online advergames. 2019. 125-50. ISBN: 9781522560647

4. Lexico. Celebrity defintion [Internet; cited 2019 October 4]. Available from: https://www.lexico.com/en/definition/celebrity.

5. Sun C-T, Ye S-H, Wang Y-J. Effects of commercial video games on cognitive elaboration of physical concepts. Comput Educ. 2015;88:169-81.[doi: 10.1016/j.compedu.2015.05.002].

6. Consumer-generated advertising: definition & examples [Internet]. Date unknown [cited 2019 October 11]. Available from: https://study.com/academy/lesson/consumer-generated-advertising-definition-examples.html.

7. Bhasin H. What is corporate branding? [Internet]. 2018 [cited 2019 October 4]. Available from: https://www.marketing91.com/corporate-branding/.

8. Petty RE, Cacioppo JT. The Elaboration Likelihood Model of Persuasion. 1986. 123-205. ISBN:978-1-4612-4964-1

9. Klassen KM, Douglass CH, Brennan L, Truby H, Lim MSC. Social media use for nutrition outcomes in young adults: a mixed-methods systematic review. Int J Behav Nutr Phys Act. 2018;15(1):70. [doi:10.1186/s12966-018-0696-y]. PMID:30041699.

10. What is Facebook? [Internet]. Lifewire; 2019 [cited Mar 2019 March 1. Available from: https://www.lifewire.com/what-is-facebook-3486391

11. Baddeley M. Herding, social influence and economic decision-making: socio-psychological and neuroscientific analyses. Philos Trans R Soc Lond B Biol Sci. 2010;365(1538):281-90. [doi:10.1098/rstb.2009.0169]. PMID:20026466.

12. Thomson M. Human brands: investigating antecedents to consumers’ strong attachments to celebrities. J Mark. 2006;70(3):104-19.[doi:10.1509/jmkg.70.3.104].

13. Lup K, Trub L, Rosenthal L. Instagram #instasad?: exploring associations among instagram use, depressive symptoms, negative social comparison, and strangers followed. Cyberpsychol Behav Soc Netw. 2015;18(5):247-52. [doi:10.1089/cyber.2014.0560].

14. Miriam J. Metzger, Andrew J. Flanagin, Keren Eyal, Daisy R. Lemus & Robert M. Mccann. Credibility for the 21st Century: Integrating perspectives on source, message, and media credibility in the contemporary media environment, ICA. 2003;27(1):293-335. [doi:10.1080/23808985.2003.11679029].

15. Senft T. Camgirls: celebrity and community in the age of social networks: Peter Lang; 2008. 150. ISBN:9780820456942

16. NSA voluntary register of nutritionists [Internet]. Australia: The Nutrition Society Of Australia; 2019 [cited 2019 April 4]. Available from: https://nsa.asn.au/nsa-voluntary-register-of-nutritionists/.

17. Post-truth definition [Internet]. Cambridge Dictionary; 2019 [cited 2019 October 15]. Available from: https://dictionary.cambridge.org/dictionary/english/post-truth.

18. Lin X, Spence PR. Others share this message, so we can trust it? An examination of bandwagon cues on organizational trust in risk. Inf Process Manag. 2019;56(4):1559-64.[doi:10.1016/j.ipm.2018.10.006].

19. Ryan RM, Deci EL. Intrinsic and extrinsic motivations: classic definitions and new directions. Contemp Educ Psychol. 2000;25(1):54-67.[doi:10.1006/ceps.1999.1020].

20. Ergin U, Taner G. The snop effect in the consumption of luxury goods. Procedia Soc Behav Sci. 2012;62:628-37. [doi: 10.1016/j.sbspro.2012.09.105].

21. Klassen KM, Borleis ES, Brennan L, Reid M, McCaffrey TA, Lim MS. What people "like": analysis of social media strategies used by food industry brands, lifestyle brands, and health promotion organizations on Facebook and Instagram. J Med Internet Res. 2018;20(6):e10227.[doi:10.2196/10227]. PMID: 29903694.

22. Ohanian R. Construction and validation of a scale to measure celebrity endorsers' perceived expertise, trustworthiness, and attractiveness. J Advert. 1990;19(3):39-52.[doi:10.1080/00913367.1990.10673191].

23. What exactly is Twitter? And what is 'Tweeting'? [Internet]. Date unknown [cited 2019 October 4]. Available from: https://www.lifewire.com/what-exactly-is-twitter-2483331.

24. Lange P. Publicly private and privately public: social networking on YouTube. 2019 [cited 2019 October 1]. Available from: http://dx.doi.org/10.1007/978-3-658-11719-1_10.
