# Supplementary material for: Assessing the Credibility and Authenticity of Social Media Content for Applications in Health Communication: Scoping Review
Source: J Med Internet Res. 2020 Jul 23;22(7):e17296. doi: 10.2196/17296 (PMC7413282; doi:10.2196/17296)
Supplement: Multimedia Appendix 2 [file jmir_v22i7e17296_app2.docx]

## Multimedia Appendix 2: Final search strategy (Scopus database)

( ( ( TITLE-ABS-KEY ({social media} OR Instagram OR Facebook OR Twitter OR YouTube OR snapchat OR blog*^a^ OR microblog* OR {online platform*} OR {social networking sites} OR sns OR {social network*} OR "SMI" OR linkedin OR influencer* OR web2.0 ) OR TITLE-ABS-KEY ({online celebrit*} OR myspace OR {micro-celeb*} ) ) ) AND ( TITLE-ABS-KEY ( trust OR trustworthiness ) OR TITLE-ABS-KEY ( credibility OR credible OR believab* OR integrity ) OR TITLE-ABS-KEY ( authentic OR authenticity OR genuineness OR legitima* ) ) AND ( ( TITLE-ABS-KEY ( attitude* OR attach* OR perspective* OR response* ) OR TITLE-ABS-KEY ( {consumer perception} OR perception* OR views OR view OR judgement* OR judgment* ) ) ) ).

^a^Blogs were initially included in the search strategy, but were later excluded in order to focus on microblogging platforms only.
