# Supplementary material for: Assessing the Credibility and Authenticity of Social Media Content for Applications in Health Communication: Scoping Review
Source: J Med Internet Res. 2020 Jul 23;22(7):e17296. doi: 10.2196/17296 (PMC7413282; doi:10.2196/17296)
Supplement: Multimedia Appendix 3 [file jmir_v22i7e17296_app3.docx]

**Multimedia Appendix 3: Research studies assessing trust and credibility on Facebook**

| **Author, year, location [reference]** | **Theory or model used** | **n; population; age^a^ (mean, SD)/range; gender** | **Manipulation** | **Scale used** | **Key significant results^b^** |
| --- | --- | --- | --- | --- | --- |
| ***Trust*** | | | | | |
| Antoci et al., 2019, Italy [1] | Not reported | 412; Students; 24; 51% male, 49% female | Exposure to four authentic threads of uncivil (or civil) discussion | Yamagishi test of trust scale | Exposure to civility (e.g. politeness) increased trust by 1 experimental currency unit in the 'trust game' when compared to uncivil or neutral discussion (e.g. sexism, racism; *P* =.05). However, if previous interactions on Facebook were uncivil there was no change in participants’ trust compared to the control group. |
| Ardiansyah et al., 2018. Indonesia [2] | Self-Disclosure Theory, Consumer Socialisation Theory | 393; Students; 18-55; 45% male, 55% female | No manipulation | Trust scale adapted from Mothersbaugh et al. | People who had higher perceived privacy control had more trust in social media (*P*<.0001). A past negative experience made participants warier of trust and privacy issues (*P*=.01). Consumers with higher trust in social media were more likely to communicate with peers through social advertising (e.g. liking/sharing an ad or product; *P*<.001). |
| Phua et al., 2016, USA [3] | Social Capital Theory | 109; Students; 20.8 (1.2); 19% male, 81% female | Number of likes: high (n=47,801,273) or low (n=1,273). Number of friends' likes: high (n=128) or low (n=2) | Own scale used | Brand trust was higher when likes were high (*P*<.005), or when friends likes were high (*P*<.001). Friends' likes were more important in trust than overall total likes (*P*<.005). The number of likes had no direct effect on brand trust when intensity of Facebook use was controlled for (*P*=.89). |
| ***Credibility*** | | | | | |
| Borah et al., 2018, USA [4] | Source credibility, Sundar's MAIN model | Study 1: 340; Students; 19.8; 34% male, 66% female  Study 2: 552; Students; 19.1; 42% male, 58% female | Study 1: Frame type: gain or loss. Source type: expert (study 1: from CDC; study 2: from WebMD) or non-expert. Social endorsement: high (n=150) or low (n=2) | Adapted from Fico, Richardson, & Edwards, 2004; Meyer, 1988 | A gain-framed message (focusing on the benefits of exercise) was more credible than a loss-framed message (focusing on the risks of not exercising; study 1: *P*<.001; study 2: *P*<.001). The CDC and WebMD authors were seen as more credible than the unknown author (study 1: *P*<.01; study 2: *P*<.01). Number of likes did not affect credibility overall (study 1: *P*=.93; study 2: *P*=.09). However, when comparing the expert source with a high number of likes and the non-expert source with a low number of likes, the expert had higher credibility (study 1: *P*<.01; study 2: *P*<.01). |
| Lee, 2018, South Korea [5] | Source credibility, Sundar's MAIN model | 133; Students; 22.2 (1.56); Not reported | Number of followers: small (n=2) or large number (n=1,003). Text cue: less detailed answer or more detailed answer | Flanagin and Metzger | The number of followers made a statistically significant difference on believability of the answer (*P*<.05). A high number of followers increased believability. There were no significant results for trustworthiness or accuracy. |
| Lee, 2018, South Korea (Study 2) [5] | Source credibility, Sundar's MAIN model | 85; Students; 22.9 (3.25); Not reported | Number of friends of answerer: low (n= 10) or high (n=1,222) | Flanagin and Metzger | Perceived believability (*P*<.01) and trustworthiness (*P*<.05) of the answer were higher for the people that saw 1,222 friends. |
| Spence et al., 2013, USA [6] | Source credibility | 200; Students; Not reported; Not reported | Avatar ethnicity: Caucasian or African American | McCroskey and Teven source credibility scale | Significant effects were found for participant ethnicity. African American respondents rated the sources as more caring and trustworthy than the Caucasian respondents, regardless of avatar ethnicity (*P*<.001). African American participants rated the Caucasian avatar as the most credible in all conditions. African American participants rated the avatars as more trustworthy (*P*<.009) and caring (*P*<.002), irrespective of their ethnicity. |

**^a^**Age reported with as much detail as original paper provides, **^b^***P* values reported as in original papers, SD: Standard deviation, MAIN: modality, agency, interactivity, navigability, CDC: Centers for Disease Control

## References

1. Antoci A, Bonelli L, Paglieri F, Reggiani T, Sabatini F. Civility and trust in social media. J Econ Behav Organ. 2019;160:83-99.[doi:10.1016/j.jebo.2019.02.026].

2. Ardiansyah Y, Harrigan P, Soutar GN, Daly TM. Antecedents to consumer peer communication through social advertising: A self-disclosure theory perspective. J Int Adv. 2018;18(1):55-71. [doi:10.1080/15252019.2018.1437854].

3. Phua J, Ahn SJ. Explicating the ‘like’ on Facebook brand pages: The effect of intensity of Facebook use, number of overall ‘likes’, and number of friends' ‘likes’ on consumers' brand outcomes. J Mark Commun. 2016;22(5):544-59. [doi:10.1080/13527266.2014.941000].

4. Borah P, Xiao X. The importance of 'likes': The interplay of message framing, source, and social endorsement on credibility perceptions of health information on Facebook. J Health Commun. 2018;23(4):399-411. [doi:10.1080/10810730.2018.1455770]. PMID:29601271.

5. Lee SY. Effects of relational characteristics of an answerer on perceived credibility of informational posts on social networking sites: The case of Facebook. Inf Res. 2018;2(3).

6. Spence PR, Lachlan KA, Westerman D, Spates SA. Where the gates matter less: Ethnicity and perceived source credibility in social media health messages. Howard J Commun. 2013;24(1):1-16. [doi:10.1080/10646175.2013.748593].
