# Supplementary material for: Assessing the Credibility and Authenticity of Social Media Content for Applications in Health Communication: Scoping Review
Source: J Med Internet Res. 2020 Jul 23;22(7):e17296. doi: 10.2196/17296 (PMC7413282; doi:10.2196/17296)
Supplement: Multimedia Appendix 4 [file jmir_v22i7e17296_app4.docx]

**Multimedia Appendix 4: Research studies assessing trust and credibility on Twitter**

| **Author, year, location** | **Theory or model used** | **n; population; age^a^ (mean, SD)/range; gender** | **Manipulation** | **Scale to assess trust/credibility** | **Key significant results^b^** |
| --- | --- | --- | --- | --- | --- |
| ***Trust*** | | | | | |
| Lin et al., 2019, USA [1] | Bandwagon heuristics | 169; students; 19.7 (1.46), 18-25; 34% male, 66% female | Number of retweets: 40, 400, or 4000 | RAND public health disaster trust scale | There were significant differences in trust perceptions across the varying retweet conditions (*P*=.046). The people that viewed the Twitter FDA page containing 4,000 retweets were more likely to perceive lower organisational trust than the condition of 40 retweets (*P*<.05). |
| ***Credibility*** | | | | | |
| Houston et al., 2018, USA [2] | Not reported | 1211; paid online participants; 18+; 41% male, 59% female | Tweet space category: local or national emphasis. Tweet tone: non-opinionated, opinionated | Credibility scale adapted from Soh et al. and Thorson et al. | Tweet tone had an effect on Tweet credibility, with non-opinionated Tweets (written as a headline) perceived as more credible than opinionated (using humour/sarcasm; *P*<.001). Age group resulted in significant differences for credibility; with the younger group being more likely to have a positive reaction to the Tweets and find them more credible(*P*=.004). |
| Jahng et al., 2016, USA [3] | Source credibility, Social Information Processing Theory | 156; Students; 19.8; 39% male, 61% female | Gender of Tweeter: male or female. Social cues in bio: high (interests and hobbies) or low (where journalist works and lives). Interactivity: high (numerous replies to followers) or low (factual Tweets with no replies) | Adapted from: Berlo, Lemert, & Mertz, 1969; McCroskey, 1966; McCroskey, Holdridge, & Toomb, 1974 | Journalists with high interactivity (i.e. multiple replies to comments) were seen as more credible than those with lower interactivity (*P*<.001). Gender did not predict source credibility. |
| Jin et al., 2014, USA [4] | Social Capital Theory, Social Identity Theory, Source credibility | 160; students; 20.0 (1.0); 55% male, 45% female | Product: Bling H2O water or Oval vodka. Valence of Tweets: positive or negative. Number of followers: high (n=14,677,050) or low (n=4) | Ohanian's source credibility scale | A higher number of Twitter followers on the celebrity's account increased source credibility and intention to build an online friendship with the celebrity endorser for all dimensions of source credibility: physical attraction (*P*<.05), trustworthiness (*P*<.05) and competence (*P*<.01). |
| Jin et al., 2014, USA (Study 2) [4] | Social Capital Theory, Social Identity Theory, Source credibility | 157; students; 20.2 (1.1); 100% female | Number of followers: high (n=14,677,050) or low (n=4). Celebrity type: prosocial or antisocial | Ohanian's source credibility scale | Prosocial celebrities were seen to be more attractive (*P*<.001), trustworthy (*P*<.001), and competent (*P*<.001) than antisocial celebrities, indicating higher source credibility. |
| Lin et al., 2018, USA [5] | The MAIN model, Source credibility, Warranting theory | 434; students; 20.3 (5.96),18-55; 47% male, 53% female | Number of followers: 40, 400, or 4000. Profile: expert, student, or stranger | McCroskey and Teven source credibility scale | Participants viewing an FDA expert's account were more likely to perceive higher trust (*P*=0.01), competence (*P*<.001), and goodwill (*P*<.001) than those viewing a peer or stranger’s account. The highest level of trust was when participants viewed the post with 400 retweets, then 40 retweets, then 4,000 retweets (*P*=.01). Participants perceived the highest levels of source competence when viewing the 40 retweets, followed by those viewing the 400 and those with 4,000 (*P*=.01). |
| Lin et al., 2016, USA [6] | The MAIN model | 696; students; 20.9 (6.43), 18-79; 48% male, 52% female | Mock retweet number: no retweet or retweets (from peers or strangers). Message source: expert (CDC), peer (student account), or stranger | McCroskey and Teven source credibility scale | Higher credibility was assigned to risk information from an expert compared to a peer and a stranger (*P*<.001). Participants perceived lowest competence when viewing a peer's Twitter page with no retweets (*P*<.001). The highest level of perceived competence was when participants viewed the CDC page with no retweets (*P*<.001). The highest perceived trustworthiness was when participants viewed the CDC Twitter page with no retweets (*P*<.001). The highest levels of source goodwill were when participants viewed the CDC page with no retweets (*P*<.001). |
| Westerman et al., 2011, USA [7] | Social Information Processing Theory, Source credibility | 289; students; not reported; not reported | Number of followers: 70, 7000, or 70000. Ratio of follows to followers: narrow gap (follows is 90% followers) or wide gap (follows is 10% of followers) | McCroskey and Teven source credibility scale | Trustworthiness indicated an inverted u-shaped relationship with the number of followers (*P*=.02). Twitter profile owners with a narrow gap between number of followers they had and number of people they followed were perceived to be more competent than people with a wide gap (*P*=.02). No other dimensions of source credibility were significant. |
| Westerman et al., 2014, USA [8] | The MAIN model, Source credibility | 181; students; not reported; not reported | Recency of Twitter page update: fast (1 minute), medium (1 hour), slow (1 day) | McCroskey and Teven source credibility scale | Cognitive elaboration was positively correlated with all constructs of source credibility: competence (*r*=0.289, *P*<.001), goodwill (*r*=0.293, *P*<.001), and trustworthiness (*r*= 0.328, *P*<.001). The recency of updates had a positive linear relationship with cognitive elaboration; faster updates (1 minute) required more cognitive elaboration, which lead to increased credibility (*P*=.046). |

**^a^**Age reported with as much detail as original paper provides, **^b^***P* values reported as in original papers, FDA: Food and Drug Administration, MAIN: modality, agency, interactivity, navigability, CDC: Centers for Disease Control

## References

1. Lin X, Spence PR. Others share this message, so we can trust it? An examination of bandwagon cues on organizational trust in risk. Inf Process Manag. 2019;56(4):1559-64. [doi:10.1016/j.ipm.2018.10.006].

2. Houston JB, Brian Houston J, McKinney MS, Thorson E, Hawthorne J, David Wolfgang J, et al. The Twitterization of journalism: User perceptions of news Tweets. Journalism. 2018:146488491876445.[doi:10.1177/1464884918764454].

3. Jahng MR, Littau J. Interacting is believing: Interactivity, social cue, and perceptions of journalistic credibility on Twitter. Journal Mass Commun Q. 2016;93(1):38-58. [doi:10.1177/1077699015606680].

4. Jin S-AA, Phua J. Following celebrities’ Tweets about brands: The impact of Twitter-based electronic word-of-mouth on consumers’ source credibility perception, buying intention, and social identification with celebrities. J Advert. 2014;43(2):181-95. [doi:10.1080/00913367.2013.827606].

5. Lin X, Spence PR. Identity on social networks as a cue: Identity, retweets, and credibility. Commun Stud. 2018;69(5):461-82. [doi:10.1080/10510974.2018.1489295].

6. Lin X, Spence PR, Lachlan KA. Social media and credibility indicators: The effect of influence cues. Comput Human Behav. 2016;63:264-71. [doi:10.1016/j.chb.2016.05.002].

7. Westerman D, Spence PR, Van Der Heide B. A social network as information: The effect of system generated reports of connectedness on credibility on Twitter. Comput Human Behav. 2012;28(1):199-206. [doi:10.1016/j.chb.2011.09.001].

8. Westerman D, Spence PR, Van Der Heide B. Social media as information source: Recency of updates and credibility of information. J Comput Mediat Commun. 2014;19(2):171-83. [doi:10.1111/jcc4.12041].
