# Supplementary material for: Assessing the Credibility and Authenticity of Social Media Content for Applications in Health Communication: Scoping Review
Source: J Med Internet Res. 2020 Jul 23;22(7):e17296. doi: 10.2196/17296 (PMC7413282; doi:10.2196/17296)
Supplement: Multimedia Appendix 5 [file jmir_v22i7e17296_app5.docx]

**Multimedia Appendix 5: Research studies assessing credibility on both Facebook and Twitter**

| **Author, year, location** | **Theory or model used** | **n; population; age^a^ (mean, SD)/range; gender** | **Manipulation** | **Scale to assess credibility** | **Key significant results^b^** |
| --- | --- | --- | --- | --- | --- |
| Shen et al., 2019, USA [1] | Source credibility, The MAIN model | 3476; paid online participants; 34.7 (11.16), 20-87; 55% men, 45% women | Source credibility: high or low. Source and media type: website, social media individual account, or social media organisation account. Intermediary: no trust (e.g. stranger), high trust (e.g. Bill Gates), or low trust (e.g. Buzzfeed). Bandwagon: high (e.g. 64,540 favourites, 26,361 retweets) or low (e.g. 5 favourites, 3 retweets) | Flanagin and Metzger's scale of message credibility online | Differences for credibility judgements were evident for each type of picture (6 images), with the bridge collapse being the most credible (*P*<.01). People with photography skills found the images less credible than those without skills (*P*<.001). People with greater levels of internet skills thought the images were less credible (*P*=.01). People who supported the issue in the photo thought the overall credibility of the image was higher (*P*<.001). Frequent Twitter users saw images as less credible compared to those that used twitter less (*P*=.02). Bandwagon cues did not impact credibility (*P*=.85) |
| Yilmaz et al., 2016, USA [2] | Source credibility | 257; students; 20.5 (1.73), 18-24; 55% male, 45% female | Twitter: personalised Tweets, depersonalised Tweets. Facebook: personalised status updates, depersonalised status updates | Adapted from McCroskey scale | The personalised status updates on Facebook were seen as more competent than the personalised Tweets (*P*=.007). Depersonalised Tweets (i.e. factual and data-based) were more trustworthy than the source of depersonalised status updates on Facebook. Personalised Facebook updates were more trustworthy than personalised Tweets (*P*=.001). |

**^a^**Age reported with as much detail as original paper provides, **^b^***P* values reported as in original papers, MAIN: modality, agency, interactivity, navigability.

## References

1. Shen C, Kasra M, Pan W, Benefield G, Malloch Y, O'Brien J. Fake images: The effects of source, intermediary, and digital media literacy on contextual assessment of image credibility online. SSRN. 2019. [doi:10.2139/ssrn.3234129].

2. Yilmaz G, Quintero Johnson JM. Tweeting facts, Facebooking lives: The influence of language use and modality on online source credibility. Commun Res Rep. 2016;33(2):137-44. [doi:10.1080/08824096.2016.1155047].
