# Supplementary material for: Assessing the Credibility and Authenticity of Social Media Content for Applications in Health Communication: Scoping Review
Source: J Med Internet Res. 2020 Jul 23;22(7):e17296. doi: 10.2196/17296 (PMC7413282; doi:10.2196/17296)
Supplement: Multimedia Appendix 6 [file jmir_v22i7e17296_app6.docx]

**Multimedia appendix 6: Research studies assessing credibility on Instagram**

| **Author, year, location** | **Theory or model used** | **n; population; age^a^ (mean, SD)/range; gender** | **Manipulation** | **Scale to assess credibility** | **Key significant results^b^** |
| --- | --- | --- | --- | --- | --- |
| Colliander et al., 2018, Sweden [1] | Not reported | 225; paid online participants; 26; 30% male, 70% female | Snapshot aesthetic (e.g. candid) or studio aesthetic photos (e.g. formal) | Adapted from Mackenzie and Lutz brand credibility scale | Credibility was higher in the snapshot aesthetic (i.e. pictures that could be taken by the average consumer) experiment compared to the studio aesthetic (i.e. professional photoshoot; *P*<.001). |
| De Veirman et al., 2019, USA [2] | Not reported | 355; paid online participants; 32.6 (8.29); 45% male, 55% female | Sponsorship disclosure: material, financial, no compensation, or not disclosed. Message sidedness: one sided (positive review only) or two sided (both positive and negative review) | Ohanian's source credibility scale | Ad recognition (i.e. when consumers knew it was a sponsored post) was positively associated with ad scepticism (i.e. the tendency to disbelieve advertising; *r*=0.4; *P*=not reported) and negatively associated with source credibility (*r*=-0.24; *P*=not reported). Ad scepticism was negatively correlated with source credibility (*r*=-0.72; *P*=not reported). Source credibility and brand attitude were positively correlated (*r*=0.73; *P*=not reported). Mediation analysis results showed that explicitly mentioning that the post was not sponsored reduced ad recognition which, in turn, had a positive effect on scepticism (*P*<.001). Disclosing financial or material compensation lead to ad recognition which lead to increased scepticism, reducing source credibility (*P*<.001). Non-disclosed one-sided messages lead to higher credibility ratings compared to two-sided messages (*P*=.002). |
| Jin et al., 2019, not reported [3] | Source credibility, Parasocial interaction, Dual credibility model | 304; paid online participants; not reported; 100% female | Source: brand (Chanel) or influencer. Product (handbag) placement: product only or Instagram influencer with the product | Ohanian's source credibility scale | Participants that saw the influencer and the product (*Chanel* handbag) had higher perceived brand attractiveness than people exposed to product only (*P*<.001). Consumers that saw the influencer with the product indicated better attitude toward the source than the product only condition (*P*<.05). |

**^a^**Age reported with as much detail as original paper provides, **^b^***P* values reported as in original papers.

## References

1. Colliander J, Marder B. ‘Snap happy’ brands: Increasing publicity effectiveness through a snapshot aesthetic when marketing a brand on Instagram. Comput Human Behav. 2018;78:34-43. [doi:10.1016/j.chb.2017.09.015].

2. De Veirman M, Hudders L. Disclosing sponsored Instagram posts: The role of material connection with the brand and message-sidedness when disclosing covert advertising. Int J Advert. 2019:1-37. [doi:10.1080/02650487.2019.1575108].

3. Jin V, Muqaddam A. Product placement 2.0: “Do brands need influencers, or do influencers need brands?”. J Brand Manag. 2019. [doi:10.1057/s41262-019-00151-z].
