# Supplementary material for: Assessing the Credibility and Authenticity of Social Media Content for Applications in Health Communication: Scoping Review
Source: J Med Internet Res. 2020 Jul 23;22(7):e17296. doi: 10.2196/17296 (PMC7413282; doi:10.2196/17296)
Supplement: Multimedia Appendix 7 [file jmir_v22i7e17296_app7.docx]

**Multimedia appendix 7: Research studies assessing credibility on YouTube**

| **Author, year, location** | **Theory or model used** | **n; population; age^a^ (mean, SD)/range; gender** | **Manipulation/intervention** | **Scale to assess trust/credibility** | **Key significant results^b^** |
| --- | --- | --- | --- | --- | --- |
| English et al., 2011, USA [1] | Source credibility, Aristotelian principles | 233; students; 22.0 (2.25); 27% male, 73% female | Exposure to a video with different message appeals: ethos (expert and trustworthy source), logos (logical appeal using facts), and pathos (humorous appeal) | Adapted from McCroskey and Johnson and Kayes | The ethos appeal had the highest credibility rating, followed by the logos appeal, then the pathos appeal (*P*<.01). The higher someone’s level of cynicism, the less likely they are to consider logos appeals credible (*P*<.05). Women in the logos condition tended to find the video significantly more credible than men (the speaker was a woman in this video: *P*<.05). |
| Lee et al., 2016, USA [2] | Source credibility | 175; students; 18-48; 45% male, 55% female | Source credibility: firm-generated ad or consumer generated advertising. Personality trait (extraversion, conscientiousness, openness, agreeableness, and neuroticism): low or high based on personality test | Created own scale | Subjects with low neuroticism rated the CGA condition as more credible (*P*<.01), and subjects with high neuroticism rated the firm-generated ad as more credible (*P*<.05). Other personality traits did not have a significant effect on the credibility of advertising. In general, credibility was higher in the consumer-generated ad condition compared to the firm-generated ad condition (*P*<.05). |
| Zimmermann et al., 2018, Germany [3] | The MAIN model | 147; students; 30.4 (9.85), 18-61; 41% male, 59% female | Thematic reference of sidebar: similar or unrelated content to the main video. Linguistic style: conversational or formal | Matthes and Kohring | Participants judged credibility differently depending on the topic of the nutrition myths (*P*<.001). Interaction effects indicated that the linguistic style affected trustworthiness: someone who is more conversational in a context that had similar videos in the sidebar was seen as more trustworthy (*P*=.04). Linguistic style and sidebar content did not have a main effect on credibility. |

**^a^**Age reported with as much detail as original paper provides, **^b^***P* values reported as in original papers, CGA: consumer-generated advertising, MAIN: modality, agency, interactivity, navigability.

## References

1. English K, Sweetser KD, Ancu M. Youtube-ification of political talk: An examination of persuasion appeals in viral video. Am Behav Sci. 2011;55(6):733-48. [doi:10.1177/0002764211398090].

2. Lee JK, Lee S-Y, Hansen SS. Source credibility in consumer-generated advertising in Youtube: The moderating role of personality. Curr Psychol. 2017;36(4):849-60. [doi:10.1007/s12144-016-9474-7].

3. Zimmermann M, Jucks R. With a view to the side: Youtube’s sidebar and Youtuber’s linguistic style as hints for trust-related evaluations. Int J Hum Comput Interact. 2018:1-13. [doi:10.1080/10447318.2018.1519165].
